# Disturbance of consciousness due to hyperammonemia and lactic acidosis during mFOLFOX6 regimen: Case report

**DOI:** 10.1097/MD.0000000000021743

**Published:** 2020-08-14

**Authors:** Masafumi Fukuda, Masakazu Nabeta, Takanori Muta, Tomonori Cho, Yutaka Shimamatsu, Yasutaka Shimotsuura, Kei Fukami, Osamu Takasu

**Affiliations:** a Advanced Emergency and Critical Care Center, Kurume University Hospital; b Department of Emergency and Acute Intensive Care Medicine; c Division of Gastroenterology; d Division of Nephrology, Department of Medicine, Kurume University School of Medicine, Kurume, Fukuoka, Japan.

**Keywords:** chronic kidney disease, FOLFOX therapy, hyperammonemia, lactic acidosis

## Abstract

**Introduction:**

FOLFOX therapy is the main chemotherapy regimen for colorectal cancer. Peripheral neuropathy, hematotoxicity, and digestive symptoms are known to be the most frequent adverse events. Hyperammonemia and lactic acidosis rarely occur simultaneously during treatment with FOLFOX therapy; the number of case reports is limited worldwide. We report a case of disturbance of consciousness, considered to be caused by hyperammonemia and lactic acidosis that occurred during treatment with mFOLFOX6 therapy that was administered as postoperative adjuvant treatment for rectal cancer.

**Patient concerns:**

This case was of a 71-year-old man who had been receiving oral treatment for chronic kidney disease and diabetes mellitus. Laparoscopic low anterior resection and artificial anal construction surgery were performed for stage III rectal cancer. As adjuvant postoperative therapy, mFOLFOX6 therapy was started but was followed by a disturbance of consciousness.

**Diagnoses:**

Results of the blood tests revealed notable hyperammonemia (ammonia level, 1,163 μg/dl) and lactic acidosis (pH 7.207; lactate, 17.56 mmol/L); however, imaging diagnosis did not reveal intracranial lesions that could cause disturbance of consciousness.

**Interventions:**

For hyperammonemia, branched-chain amino acid agents and Ringers solution supplementation were administered. For acidosis, 7% sodium hydrogen carbonate was administered as treatment.

**Outcomes:**

The disturbance of consciousness improved within 12 hours of initiating the treatment, and the patient was discharged with no sequelae on 7th day after hospitalization.

**Conclusion:**

In patients with chronic kidney disease, FOLFOX regimen may confer risks of hyperammonemia and lactic acidosis.

## 1 Introduction

In recent years, a series of effective chemotherapeutic drugs for colorectal cancer have improved greatly in terms of treatment outcomes.^[1]^ Among these drugs, 5-fluorouracil (5-FU), since its development in 1957, has become a key drug in the systemic chemotherapy, which is effective for the treatment of colorectal cancer.^[2]^ FOLFOX therapy, which is a combined therapy with oxaliplatin, is a key regimen in chemotherapy for colorectal cancer. However, strong anti-tumor and life-prolonging effects are expected.^[3]^ Cases of hyperammonemia during FOLFOX therapy have been reported, but cases of hyperammonemia and lactic acidosis occurring simultaneously are rare. We report a case of disturbed consciousness due to hyperammonemia and lactic acidosis, considered to be caused by 5-FU administration when mFOLFOX6 therapy was administered as part of the postoperative chemotherapy for rectal cancer. Furthermore, Consent was obtained from the patient himself regarding the presentation of the case in this paper.

## 2 Case presentation

### 2.1 Clinical course and diagnosis

This case was of a 71-year-old man with a chief complaint of disturbance of consciousness. His medical history included oral treatment with 5-mg linagliptin, 5-mg mitiglinide, and 5-mg enalapril for diabetes and chronic kidney disease. A laparoscopic low anterior resection and artificial anal construction had been performed for stage IIIa rectal cancer by a previous physician. One course of mFOLFOX6 therapy (oxaliplatin with 5-FU 2400 mg/m^2^/week) was administered postoperatively, but due to mild lack of appetite and nausea, the patient had poor oral intake and reduced water consumption; therefore, central vein nutritive therapy was also started. The second course was started, and after 48 hours, oxaliplatin and 5-FU were administered by constant infusion, and 60 minutes after initiation of the second course of chemotherapy, disquietness, stertorous breathing, and a disturbance of consciousness of Glasgow Coma Scale score of approximately 7 were observed. Intracranial disease was suspected; magnetic resonance imaging (MRI) and computed tomography (CT) were performed, however, no significant abnormal findings were observed. The patient was admitted to our hospital to investigate the cause of the disturbance of consciousness and to provide treatment. The state of consciousness recorded on admission was Glasgow Coma Scale score of 8, and blood pressure was 160/110 mm Hg, pulse was 110/minute, and breathing rate was 34 breaths/minute. Tachypnea was also observed. He had no fever, and his limbs were toned. Although tonic seizures in both arms were observed, no abnormalities of the pupils were observed. Patient consent was obtained for participation in this study and for publication of this case report and laboratory data.

### 2.2 Laboratory tests

The blood test results on admission are shown in Table 1. The results of the blood gas analysis under administration of 2 L of oxygen were as follows: pH 7.207, PaO_2_ 158.7 mm Hg, PaCO_2_ 7.9 mm Hg, and HCO_3_^−^ 3.0 mmol/L. Metabolic acidosis was observed. Lactic acid and ammonia had notably high values of 17.56 mmol/L and 1,163 μg/dl, respectively. Liver function did not show notable abnormalities; however, kidney function impairment was evident based on the creatinine level of 2.51 mg/dl. No alcohol was detected, and the drug screening result was negative. His triglyceride level was low (34 mg/dl) and thiamine level had not reduced (41 ng/ml, reference range: 24–66 ng/ml). Chest radiography revealed a central catheter inserted from the right internal jugular vein but no significant abnormal findings were observed. Moreover, Head-to-abdomen CT revealed also no abnormal findings.

**Table 1:**
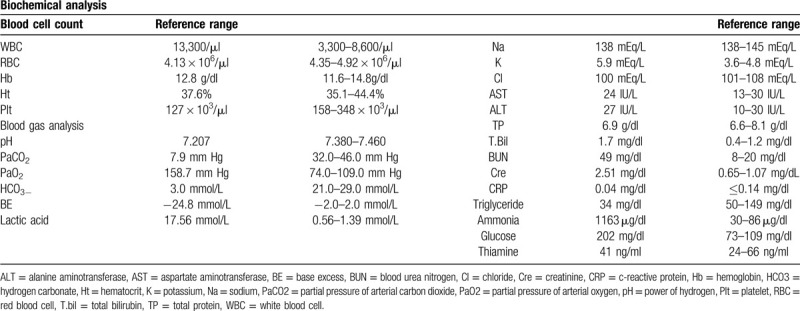
Laboratory data on admission.

### 2.3 Post-admission course

**Figure 1:**
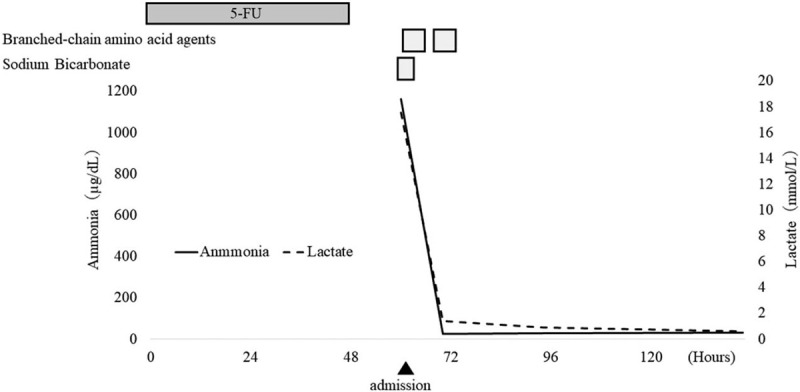
The variation trend of lactic acid and ammonia in our patients plasma after treatments. Twelve  hours after the start of treatment and the ammonia value improved to 24 μg/dl, as well as lactic acid to 1.4 mmol/L.

Based on the course until admission to our hospital and laboratory findings, hyperammonemia and lactic acidosis were suspected to be the cause of the disturbance of consciousness, and treatment was started. The clinical course after the start of treatment is shown in Figure 1. The tongue root had sunk because of the disturbance of consciousness, so a nasal airway tube was inserted. For hyperammonemia, branched-chain amino acid agents and supplementation with Ringers solution were administered. For acidosis, administration of 7% sodium bicarbonate tartaric acid was started. The disturbance of consciousness improved 12 hours after the start of treatment, and the ammonia value reduced to 24 μg/dl, and improved lactic acidosis (pH 7.456, PaO_2_ 125.4 mm Hg, PaCO_2_ 26.9 mm Hg, HCO_3−_ 18.7 mmol/L, and lactic acid 1.4 mmol/L). No sequelae was observed, the disturbance of consciousness improved, and the patient was discharged on 7th day of hospitalization.

## 3 Discussion

Peripheral neuropathy; hematotoxicities such as neutropenia, thrombocytopenia, and anemia; and digestive symptoms such as diarrhea and nausea are adverse events of mFOLFOX6 therapy for colorectal cancer, occurring at relatively high frequencies;^[4]^ however, disturbance of consciousness is rare. The reversible posterior leukoencephalopathy syndrome, which is a disturbance of consciousness accompanied by convulsions, headaches, and visual field defects, has been reported with single drug use of 5-FU^[5]^ and with FOLFOX therapy.^[6]^ However, in this case, CT/MRI did not reveal posterior cerebral white matter lesions reflecting a cerebral edema; therefore, the involvement of reversible posterior leukoencephalopathy syndrome was ruled out. Disturbance of consciousness triggered by 5-FU has 2 possible causes other than reversible posterior leukoencephalopathy syndrome.^[7]^ The first is dihydropyrimidine dehydrogenase (DPD) deficiency. Liver is the main metabolizing organ of 5-FU, and a series of pyrimidine metabolic enzymes decompose and are inactivated.^[8]^ DPD, which primarily exists in the liver, inactivates 80% to 90% of the administered 5-FU dose and decomposes it into 5-fluoro-β-alanine (FBAL), carbon dioxide, and ammonia. In other words, the disturbance of consciousness arising from a deficiency in DPD is due to the accumulation of 5-FU. Owing to an increase in a metabolite, ammonia, disturbance of consciousness due to DPD deficiency was deemed to be unlikely. The second cause is hyperammonemia. Various cases of hyperammonemia caused by 5-FU have been reported in association with various cancers.^[9–11]^ Fluoro mono acetate, a metabolite of FBAL, inhibits the tricarboxylic acid (TCA) cycle, which then reduces the level of adenosine triphosphate (ATP), thereby decreasing the metabolism via the urea cycle, resulting in an accumulation of ammonia.^[12]^ Severe hyperammonemia was observed in this case and was considered to be potential cause of disturbance of consciousness. Ammonia is a metabolite of 5-FU; thus, in theory, the level of the metabolite, ammonia, increases depending up on the dosage of 5-FU. The onset of hyperammonemia has often been reported with high-dose administrations of 5-FU (2200–2600 mg/m^2^/week).^[10]^ However, other reports have demonstrated hyperammonemia with dosage less than that in this case^[11]^; thus, we can infer that high dosage alone does not trigger hyperammonemia and that the bodys ammonia-processing capability also contributes greatly. In fact, the decomposition of ammonia occurs in the liver, but it is also known to occur in the skeletal muscles, kidneys, and brain.^[13]^ If ammonia is produced in excess, the amount of ammonia that cannot be processed by the liver will be processed by the kidneys, and in case of hyperammonemia, the kidneys reduce the production of ammonia and increase the urinary excretion of ammonia.^[14]^ Dehydration^[15]^ or kidney function impairment^[16]^ have been reported as risk factors of hyperammonemia accompanying 5-FU administration. These are considered to be a result of the reduced ammonia clearance. Although the present case had no liver function impairment, the chronic kidney disease may have been the cause of the hyperammonemia.

In this case, a significant lactic acidosis was observed along with hyperammonemia. Lactic acidosis can occur via various mechanisms,^[17]^ however majority can be classified into 2 types, types A and B, depending on the cause.^[18]^ In this case, type B rather than type A, which accompanies reduced oxygen in the tissues, was the likely contributing factor. No underlying liver impairment was found. Although the patient had diabetes mellitus, he had no notable blood sugar abnormality. Hence, liver impairment or diabetes mellitus was deemed to have no direct contribution to the lactic acidosis. Fluorouracil drug-related type B acidosis has been reported previously.^[19]^ In type B acidosis, 5-FU increases thiamine metabolism in the cells, thereby inducing thiamine deficiency.^[20]^ Initially, thiamine deficiency was considered a potential cause. This is because thiamine deficiency prevents pyruvic acid from being oxidized to acetyl-CoA, consequently, producing excessive lactic acid, thereby triggering lactic acidosis.^[21]^ However, results of the blood test on hospital admission did not reveal thiamine deficiency. Another potential cause was identified by a study reporting hypotriglyceridemia as a potential risk factor of lactic acidosis.^[7]^ Hypotriglyceridemia impairs the ATP-producing lipolysis, which aids recovery from the ATP-deficient state caused by impairment of the TCA cycle, and consequently prolongs lactic acidosis. In this case, hypotriglyceridemia was considered a contributing factor to the resulting lactic acidosis. In addition, lactic acid is mainly cleared by the liver, with partial contribution of the kidneys.^[22]^ Therefore, chronic kidney disease may have contributed to the lactic acidosis.

Here, we report an extremely rare case of hyperammonemia occurring simultaneously with lactic acidosis during mFOLFOX6 therapy following surgery for rectal cancer. Until now, the reported cases of disturbance of consciousness due to 5-FU therapy have mainly resolved through symptomatic treatments.^[7]^ In the present case, the symptomatic treatment course was good without sequelae.

## 4 Conclusion

We report a case of rectal cancer with disturbance of consciousness due to hyperammonemia and lactic acidosis during mFOLFOX6 therapy after surgery for rectal cancer. The presence of chronic kidney disease indicates the possibility of the onset of hyperammonemia and lactic acidosis even in the absence of liver impairment. Therefore, in cases with chronic kidney disease, it is important to detect complications that are life-threatening but treatable by monitoring acid bases, lactic acid, and ammonia.

## Author contributions

**Conceptualization:** Masafumi Fukuda.

**Investigation:** Takanori Muta, Tomonori Cho, Yutaka Shimamatsu.

**Methodology:** Masafumi Fukuda.

**Project administration:** Nabeta Masakazu.

**Resources:** Masafumi Fukuda.

**Supervision:** Kei Fukami, Osamu Takasu.

**Visualization:** Masafumi Fukuda, Yasutaka Shimotsuura.

**Writing – original draft:** Masafumi Fukuda.

**Writing – review and editing:** Yasutaka Shimotsuura, Osamu Takasu.
